# Tuning the metal-support interaction in the thermal-resistant Au–CeO_2_ catalysts for CO oxidation: influence of a mild N_2_ pretreatment†

**DOI:** 10.1039/c8ra07278g

**Published:** 2018-11-23

**Authors:** Yuqi Sun, Wei Liu, Miao Tian, Liguo Wang, Zhongpeng Wang

**Affiliations:** School of Water Conservancy and Environment, University of Jinan Jinan 250022 China stu_liuw@ujn.edu.cn chm_wangzp@ujn.edu.cn

## Abstract

Pretreatment is very important for altering the catalytic properties of the supported noble metal catalysts in many heterogeneous reactions. In this study, a simple and mild pretreatment with N_2_ has been reported to re-activate the Au–CeO_2_ catalysts that were prepared by a deposition–precipitation method followed by calcination at 600 °C. Upon N_2_ pretreatment at 200 °C, the metal-support interaction between Au nanoparticles (NPs) and CeO_2_ was observed with the evidence of particular coverage of Au nanoparticles by CeO_2_, electronic interactions and changes in CO adsorption ability. As a result, the CO oxidation activity of the pretreated Au–CeO_2_ catalysts largely improved compared with those without any pretreatment and even with those subjected to H_2_ and O_2_ pretreatments. N_2_ pretreatment also makes the Au NPs more resistant to sintering at high temperature. Furthermore, this mild pretreatment strategy can provide a potential approach to improve the thermal stability of other supported noble metal catalysts.

## Abstract 2

The degree of encapsulation for Au–CeO_2_ catalysts was identical to the catalysts exhibiting metal-support interaction, which improved the CO oxidation activity.

## Graphical abstract

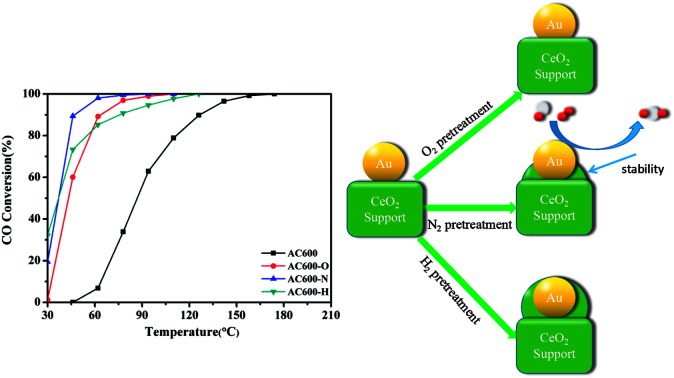

## 1. Introduction

Au nanoparticles (NPs) as the active species with superior catalytic activities have been widely used in many heterogeneous catalytic reactions. For example, the modification of Au NPs has attracted considerable attention during the last two decades in order to further develop the potential applications in the industrial removal of automobile exhausts, particularly evolved during the oxidation of CO.^1–3^ The highly active Au NPs supported on oxides or non-oxides, such as Au/TiO_2_,^4^ Au/HAP,^5^ Au/Co_3_O_4_ (ref. 6) and Au/CeO_2_,^7^ have emerged as one of the best candidates for CO oxidation. However, Au NPs are thermodynamically unstable and tend to be easily sintered and inactivated at temperatures above 400 °C.^8^ Since then, numerous studies have been extensively reported on active supported Au catalysts with the sintering-resistant property. As a result, it has been confirmed that the catalytic activity strongly depends on the support effect of oxides/non-oxides, the size effect of Au NPs, the characteristics of the oxygen species, as well as the metal-support interactions.^9–11^

Amongst all the attributes of supported noble metal catalysts, the introduction of the metal-support interactions is a particular perspective to improve the stability of Au NPs. Several strategies have been developed with the SMSI approach to stabilize Au NPs for the CO oxidation processes. Gu *et al.* reported that the Au@CeO_2_ yolk–shell structures exhibited good catalytic stability owing to the protection of the CeO_2_ shell.^12^ Zhan *et al.* constructed a sacrificial carbon layer on the Au–TiO_2_ surface with the introduction of polydopamine and found that the interactions between TiO_2_ and Au NPs remarkably enhanced, while the carbon layers could be removed through oxidative calcination in air.^13^ Tang *et al.* demonstrated that a classical SMSI for Au/TiO_2_ could be observed upon harsh high-temperature redox pretreatments, and in the SMSI state, the stability of Au/TiO_2_ toward CO oxidation drastically improved.^14^ However, it is necessary to develop a simple and mild method to re-activate the Au NPs that are calcined at high temperature.^15^

Herein, inspired by developments achieved in other studies and our previous study, a mild inert pretreatment with N_2_ was utilized to achieve metal-support interactions in the Au–CeO_2_ catalysts and to trace whether the active sites can be re-exposed in the CO oxidation reaction.^16–18^ Hence, in our study, the Au–CeO_2_ spheres calcined at 600 °C and utilized as model catalysts after pretreatment in different atmospheres (O_2_, N_2_, and H_2_) at 200 °C. Various physicochemical characterizations were employed to elucidate the effect of the pretreatments on the catalytic performance of CO oxidation. We found that the samples pretreated in N_2_ possessed the best catalytic activities at lower temperatures compared with those without any pretreatments and even O_2_ and H_2_ pretreatments. The key to success was the achievement of metal-support interaction with the assistance of N_2_ pretreatment, as demonstrated by the particular coverage of Au nanoparticles by CeO_2_, the electron transfer and the changes in CO adsorption ability. This new strategy is expected to reactivate Au NPs sintered in the calcination process with the introduction of metal-support interactions, and can be extended to other supported noble metal catalysts.

## 2. Experimental

### A Materials

Cerium nitrate hexahydrate (99.9%, Ce (NO_3_)_3_·6H_2_O) was purchased from Tianjin Kermel Co. Ltd. Polyvinyl pyrrolidone (PVP, K30), ethylene glycol and chloroauric acid (HAuCl_4_·4H_2_O, 99%) were purchased from Sinopharm Chemical Reagent Co. Ltd. All reagents were used without further purification. Deionized water and absolute alcohol were used throughout.

### B The synthesis of Au–CeO_2_ samples

CeO_2_ nanospheres were synthesized using a method with some modification according to our previous study. Initially, 1.0 g Ce (NO_3_)_3_·6H_2_O and 0.4 g (PVP) were dissolved in 30 mL ethylene glycol and 2 mL distilled water. Then, the mixture was stirred for 20 min. The resulting clear solution was transferred to a 100 mL-Teflon-lined autoclave and heated at 160 °C for 8 h. When the autoclave was cooled to room temperature, the mauve products were collected and washed three times with deionized water and ethanol, in sequence. The CeO_2_ products were dried at 60 °C in an oven overnight.

The Au–CeO_2_ samples were prepared by a deposition–precipitation method. Initially, 1 mL of HAuCl_4_·4H_2_O (0.024 mol L^−1^) was added to 9 mL deionized water, with the solution pH adjusted to 9 by adding NaOH (0.1 mol L^−1^). After stirring for 20 min, 10 mL CeO_2_ precursor solution (0.145 mol L^−1^) was added into the above solution. pH of the mixture solution was maintained at ∼9 for 1 h by addition of NaOH (0.1 mol L^−1^) at room temperature. Then, the mixture was heated to 60 °C and stirred for 1 h. The products were collected and washed with deionized water and dried at 60 °C for 12 h. The sample was calcined in a muffle furnace at 600 °C for 3 h. Following this, the catalysts were pretreated at 200 °C for 0.5 h in different atmospheres, namely, 10% O_2_ in He, 99.9% N_2_, and 5% H_2_ in N_2_ and denoted as AC600-O, AC600-N and AC600-H, respectively. In addition, the samples without any pretreatments were denoted as AC600.

### C Characterization

Phase purity of the samples was examined by using a Bruker D8 advance X-ray diffractometer (XRD) with Cu-Kα radiation (*λ* = 0.15406 nm) in the 2*θ* range from 10° to 90°. The micro-structure and morphology of the products were characterized using an X-ray spectrometer (X-MAX-50) and a field-emission scanning electron microscope (TEM, JEM-2100F) equipped with energy-dispersive X-ray spectroscopy (EDS). The existence of surface elements and their valence states were confirmed by X-ray photoelectron spectroscopy (XPS, Thermo Scientific Escalab 250Xi). The CO adsorption was determined by *in situ* diffuse reflectance infrared Fourier transform spectra (DRIFTS, Nicolet IS50). CO (0.2% CO/N_2_, flow rate: 50 mL min^−1^) was introduced into the catalyst at RT (30 °C) for 20 min and the spectra were recorded until there were no variations observed. Then, the spectra for CO adsorption under the purge of He (50 mL min^−1^) at room temperature were also collected.

### D Catalytic tests

The catalytic activity test was performed using a temperature-programmed oxidation (TPO) technology in a fixed-bed quartz reactor with length of 240 mm and inner diameter of 7 mm. Initially, 50 mg of catalyst was sieved into a 40-80 mesh and used without any dilution. Prior to the reaction, the total flow rate of the reaction gas was 100 mL min^−1^ with a composition of 0.2% CO (balanced with He) and 5% O_2_ (balanced with He), resulting in a space velocity (SV) of 120 000 mL g_cat_^−1^ h^−1^. After pretreatments, the samples were heated from 30 °C at the rate of 4 °C min^−1^. The products were detected with an online gas chromatograph (GC-2080) equipped with a thermal conductivity detector (TCD). The stability tests were measured with the same reactor and the same feed gas as described above at 70 °C.

Consecutive cycling tests were performed in the same reactor and with the same feed gas. Prior to each cycle, the AC600, AC600-O, AC600-N and AC600-H samples were pretreated at 200 °C for 0.5 h under identical atmospheres (10% O_2_ in He; 99.9% N_2_; 5% H_2_ in N_2_). After pretreatments, the samples were heated from 30 °C at the rate of 4 °C min^−1^.

## 3. Results and discussion

**Fig. 1:**
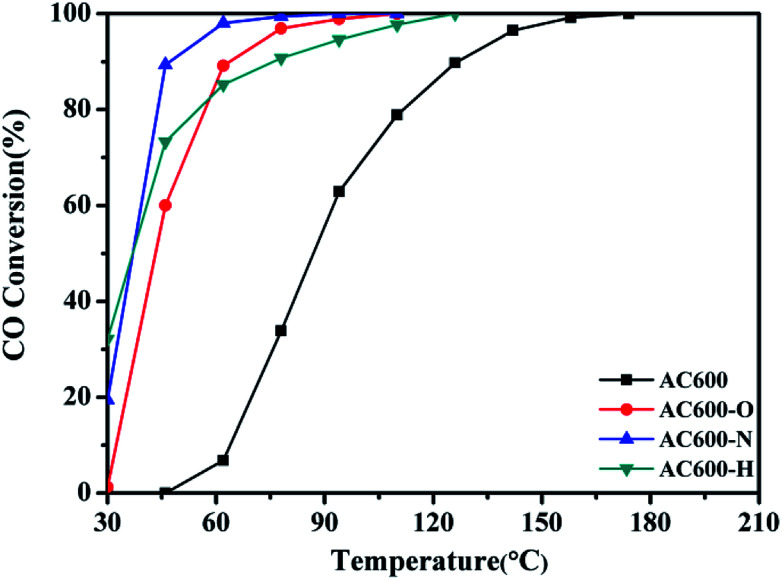
Catalytic activities of Au–CeO_2_ pretreated in different atmospheres.

CeO_2_ nanospheres were synthesized according to the method described in our previous report and the Au nanoparticles were loaded on the CeO_2_ using the DP method.^17^ CO oxidation was used as a typical probe reaction to investigate the relation between the different pretreatment conditions and the catalytic properties of the Au–CeO_2_ catalysts. Fig. 1 describes the CO conversion profiles of the various Au–CeO_2_ samples calcined at 600 °C temperatures after different pretreatments. In order to compare the catalytic activities of the abovementioned catalysts, *T*_50_ and *T*_100_ (temperatures for 50% and 100% CO conversion, respectively) of different samples were measured (Table S1†). The Au–CeO_2_ sample without any subsequent pretreatments has lower CO conversion. However, before the catalytic process, introducing different pretreatments to the samples caused a significant enhancement in the catalytic activity (Fig. 1). In particular, the AC600-N catalyst had better catalytic activities than the AC600-O and AC600-H, which had the *T*_50_ of 37 °C, while the value of *T*_100_ was about 94 °C. Notably, the initial temperature of CO conversion in AC600-N sample was 20 °C.

**Fig. 2:**
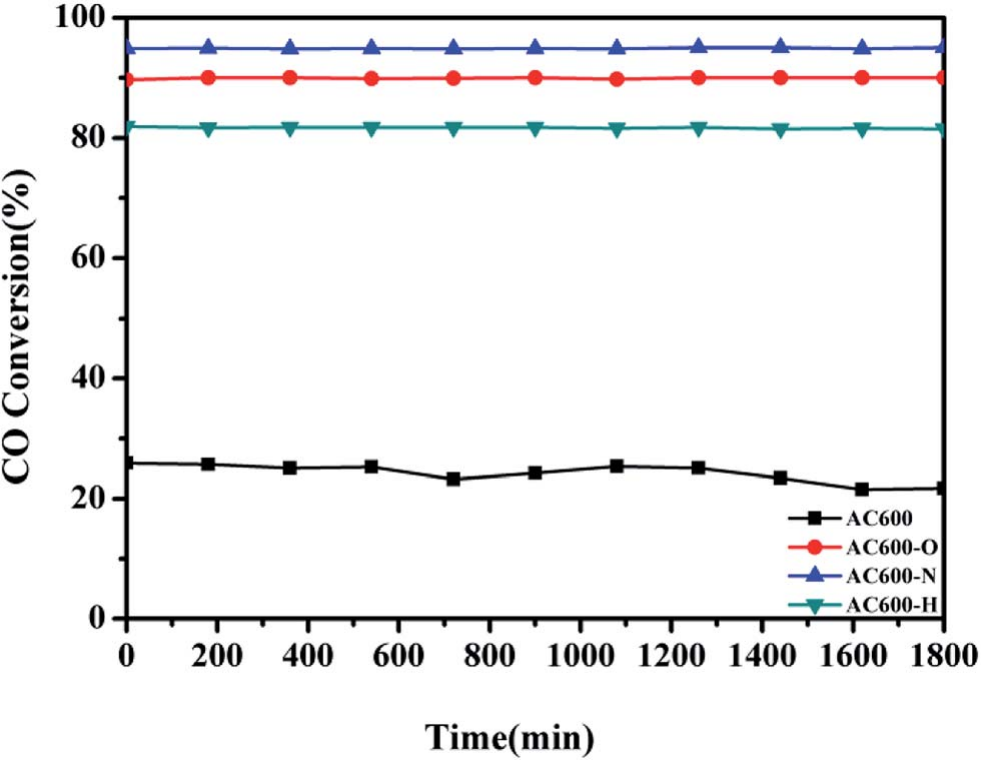
Stability tests of the Au–CeO_2_ pretreated in different atmospheres at 70 °C for CO oxidation.

Additionally, the temporal evolution profiles for CO oxidation over the differently pretreated AC600 samples (reaction at 70 °C for 1800 min) are illustrated in Fig. 2. There was no significant deactivation in CO conversion over AC600 after different pretreatments, demonstrating its sintering-resistant catalytic performance. Overall, it can be found that the pretreatment in different atmospheres (N_2_, O_2_, and H_2_) could improve the CO oxidation activity of Au–CeO_2_ samples calcined even at 600 °C. Very interestingly, the samples pretreated in mild N_2_ atmosphere have the most superior catalytic activities.

**Fig. 3:**
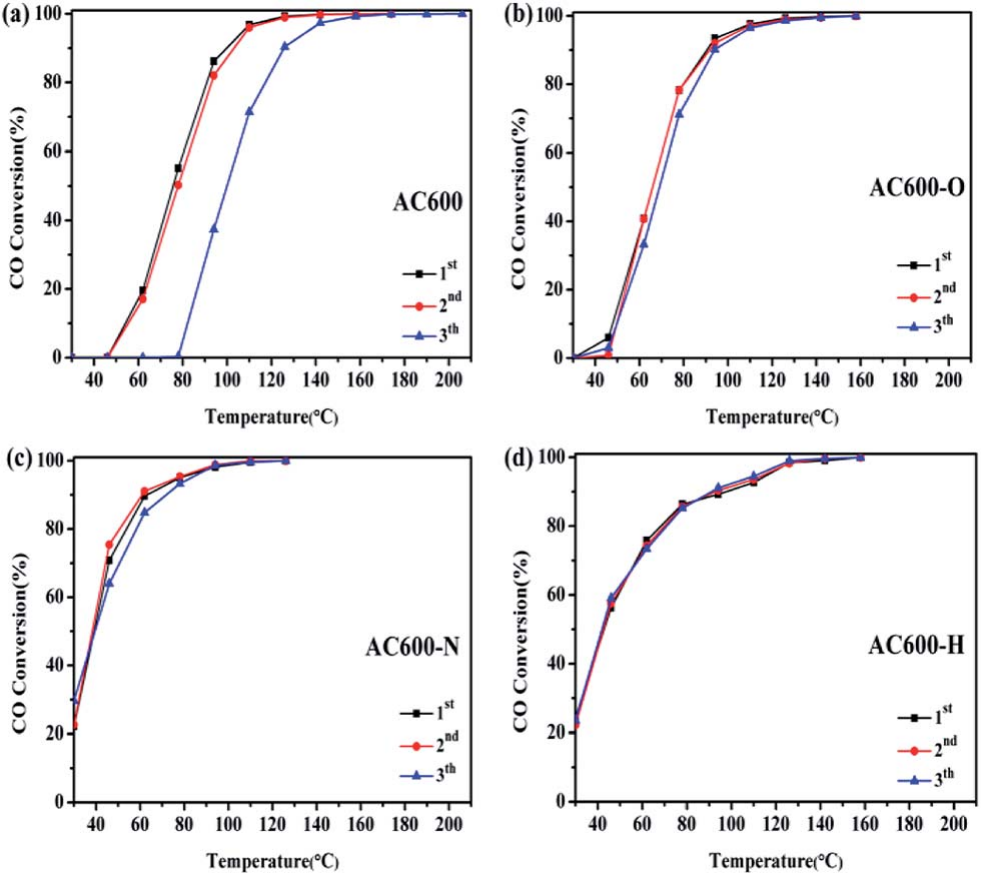
Consecutive cycles of CO oxidation on different samples ((a) AC600; (b) AC600-O; (c) AC600-N; (d) AC600-H).

Furthermore, in order to investigate the thermal stability of these samples, catalytic tests are performed in three consecutive cycles (Fig. 3). For AC600, a significant reduction of CO oxidation activity occurred rapidly in the third run. In contrast, the CO oxidation activities of the AC600-O, AC600-N and AC600-H samples remained nearly constant even after the third run. This finding further demonstrates that the pretreatment conditions can be suitable to stabilize the Au NPs after calcination.

**Fig. 4:**
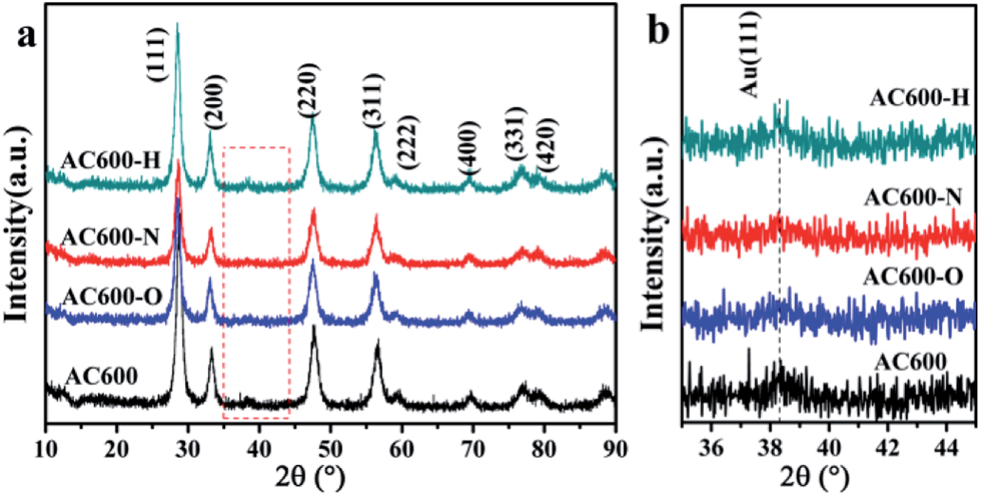
(a) XRD patterns of different samples; (b) the enlargement of the box in (a).

**Fig. 5:**
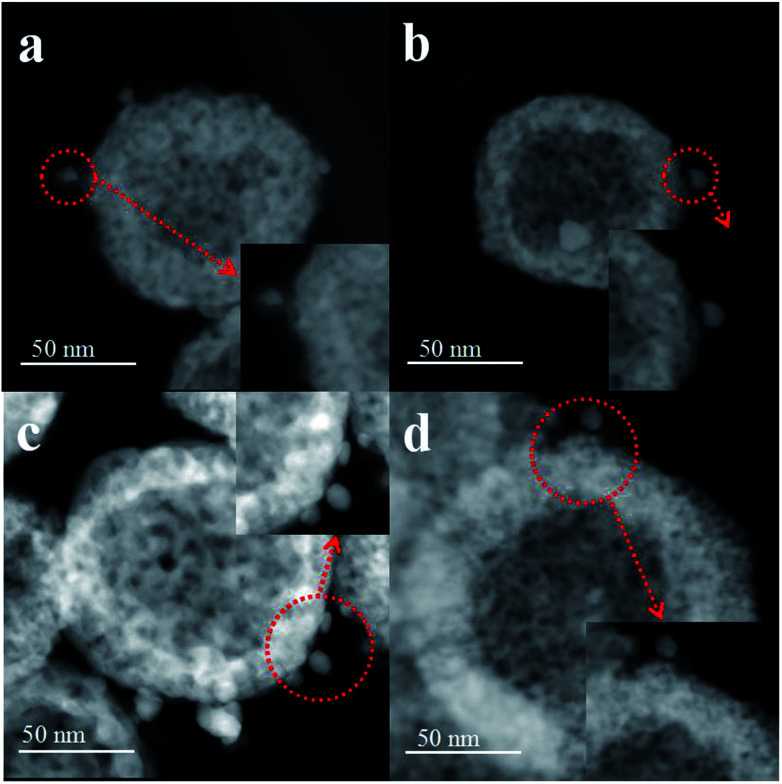
STEM images of the samples Au–CeO_2_ pretreated in different atmospheres ((a) unpretreated; (b) O_2_; (c) N_2_; (d) H_2_).

**Fig. 6:**
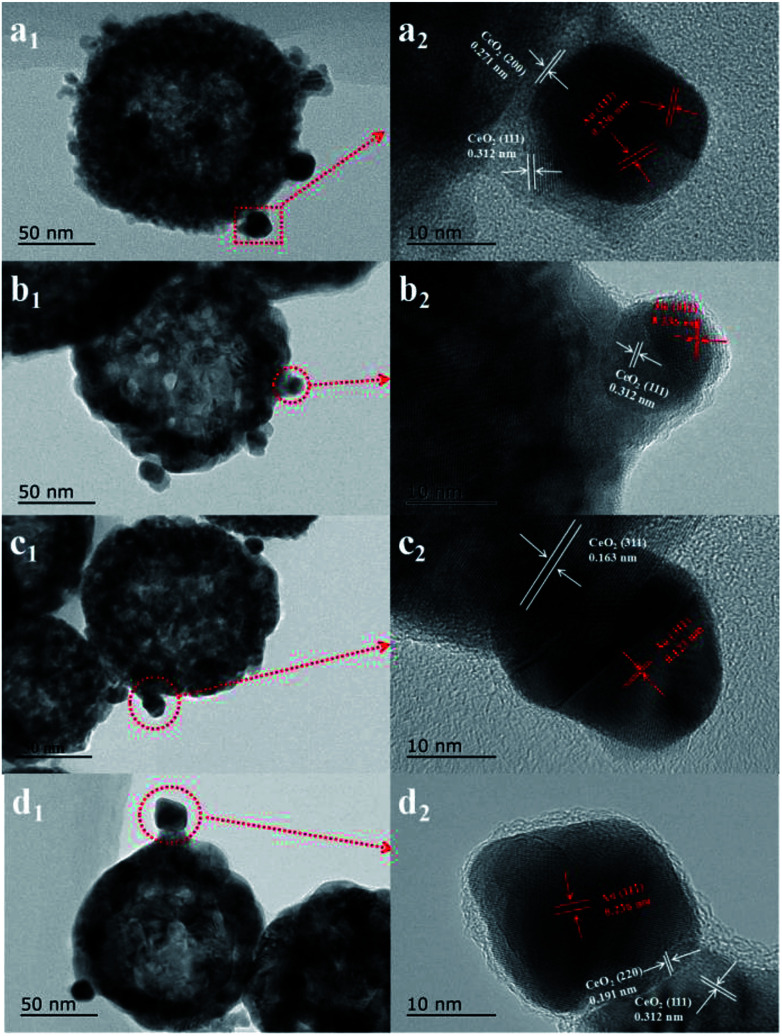
High-resolution transmission electron microscope (HRTEM) images of (a) AC600, (b) AC600-O, (c) AC600-N and (d) AC600-H samples.

First, the AC600 samples with different pretreatments were examined by powder X-ray diffraction (XRD) measurements. The pure fluorite cubic CeO_2_ phases (JCPDS no. 34-0394) for all the AC600 samples are verified in Fig. 4. Additionally, a weak diffraction peak located at 38.2° is observed for all samples, which is characteristic of Au NPs. Furthermore, the EDS results (Fig. S1†) showed that the molar ratio of Au/Ce is around 1.67 at%, which is similar to the theoretical amount (1.66%), indicating high dispersion of Au NPs on the CeO_2_ support. To further identify the microscopic structures of Au–CeO_2_ with or without any pretreatment, high-resolution transmission electron microscopy (HRTEM) was used to examine the samples. STEM characterization gives direct observation of the Au NPs and the CeO_2_ support (Fig. 5 and S3†), showing that the Au NPs were successfully attached on the surface of the porous CeO_2_ nanospheres having a particle size of approximately 110 nm. Significantly, for the AC600 sample, the diameter distribution of Au NPs was at 6.0–14.0 nm with a mean size of 9.1 nm (Fig. S2 and Table S2†). After different pretreatments, the average diameter of the Au NPs increased in the following order: AC600-O (10.1 nm) < AC600-N (10.3 nm) < AC600-H (11.2 nm). The results presented here confirm that the pretreatment conditions effectively stabilize the Au NPs and could provide a good environment to study the relation between the pretreatments and catalytic performance. Moreover, the HRTEM images of the single Au–CeO_2_ nanosphere are shown in Fig. 6. For the AC600 sample, the lattice fringes on the CeO_2_ surface of ∼0.236 nm were consistent with the (111) crystal plane of metallic Au, while that of ∼0.312 nm and ∼0.271 nm were respectively in agreement with (111) and (200) crystal plane of CeO_2_ (Fig. 5a). Similar detection for the lattice fringes of Au and CeO_2_ for AC600-O, AC600-N and AC600-H are displayed in Fig. 6b–d. As shown in Fig. 6, Au NPs on the AC600 sample without any pretreatment were naked, which is consistent with the AC600-O sample. After H_2_ pretreatment, Au NPs covered by CeO_2_ were observed on the AC600-H sample. Interestingly, naked and covered Au NPs coexist on AC600-N samples. According to the element mapping analysis (Fig. S3†), there is varied coverage of the CeO_2_ support on the Au NPs, which is a typical characteristic of the metal-support interaction phenomenon.^5^

**Fig. 7:**
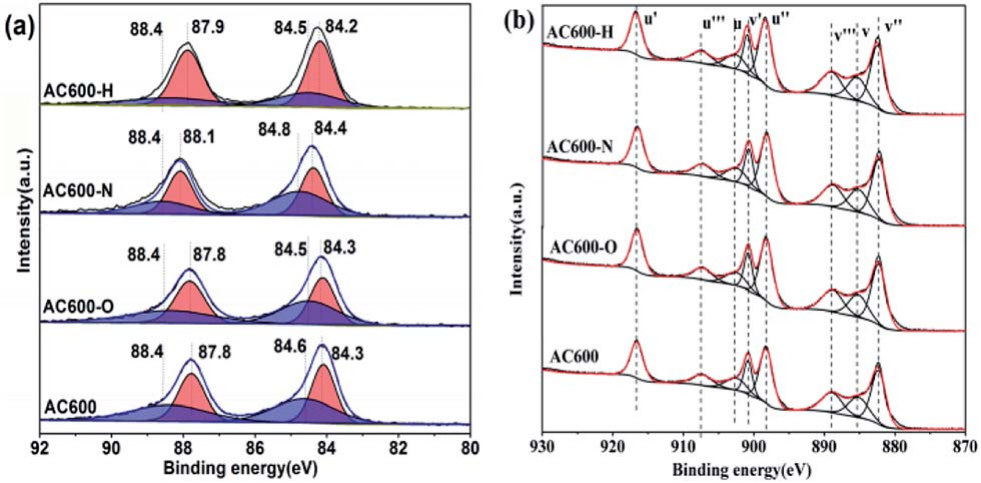
XPS spectra of AC600, AC600-N, AC600-O and AC600-H (a) Au 4f; (b) Ce 3d.

X-ray photoelectron spectroscopy (XPS) experiments were conducted in order to confirm whether there is a change in the valence states of the elements in the AC600 samples after different pretreatments. In Fig. 7a, the Au 4f spectra shows extremely widened peaks that represent different electronic states for Au species. After curve fitting, the Au 4f peaks with binding energies of about 84.3 and 87.8 eV in AC600 sample without any pretreatments were attributed to the presence of Au^0^, while two weak BE peaks at 88.4 and 84.6 eV indicate the existence of the Au^δ+^ species.^19^ After different pretreatments, these characteristic peaks were still present in the samples. However, a significant change was observed: the atomic ratio of the Au^δ+^ species decreased from 49.3% (unpretreated) to 48.2%, 23.8% and 28.7% for AC600-O, AC600-H and AC600-N samples, respectively (Table S3†). The reduction of the Au^δ+^ species in XPS indicates that the Au species are partially reduced during the treatment. The metallic Au^0^ NPs represent the active Au species, and show better CO oxidation activity.^20^ Hence, the increase of Au^0^ in AC600-N, AC600-O and AC600-H (Table S3†) provides increased catalytic performance compared with that of AC600. Additionally, a weak shift of the BE peaks at 84.4 eV to 84.2 eV is observed in the AC600-N sample. The above results imply that the Au NPs become electron-rich after the N_2_ and H_2_ pretreatments.^13^ In the Ce 3d spectra, the peaks marked as u′ (916.6 eV), v′ (900.8 eV), u′′ (898.3 eV), v′′ (882.4 eV), u′′′ (907.4 eV) and v′′′ (889.1 eV) correspond to the Ce^4+^ state, whereas those denoted as u (903.2 eV) and v (885.3 eV) are assigned to Ce^3+^.^21,22^ In Table S4,† the results of the primary binding energies of Ce 3d achieved by the XPS quantitative analysis, are reported for the differently pretreated samples. The similar features of the Ce 3d spectra for these samples are demonstrated in Table S4.† In our study, the pretreatment atmospheres, including the N_2_ pretreatments, could be a powerful tool to alter the electron interactions between Au NPs and CeO_2_. Overall, the strong electron transfers are created by N_2_ or H_2_ pretreatment, resulting in electron-rich Au.

It has been reported that the surface hydroxyl (OH^−^) group has a significant effect on the activity of supported Au catalysts.^23^ As shown in Fig. S3,† the O 1s XPS spectra showed a wide peak. After fitting analysis, the band at ∼529.5 eV is related to the lattice oxygen, whereas those at ∼531.5 eV and ∼533.7 eV are ascribed to OH^−^ groups and adsorbed H_2_O, respectively.^24^ As shown in Table S5†, the content of all samples are 64–69% for lattice oxygen, 24–28% for OH^−^ group and ∼7% for adsorption of H_2_O. Therefore, the similar amount of OH^−^ group species in these samples indicates that the effect of OH^−^ group is negligible.

**Fig. 8:**
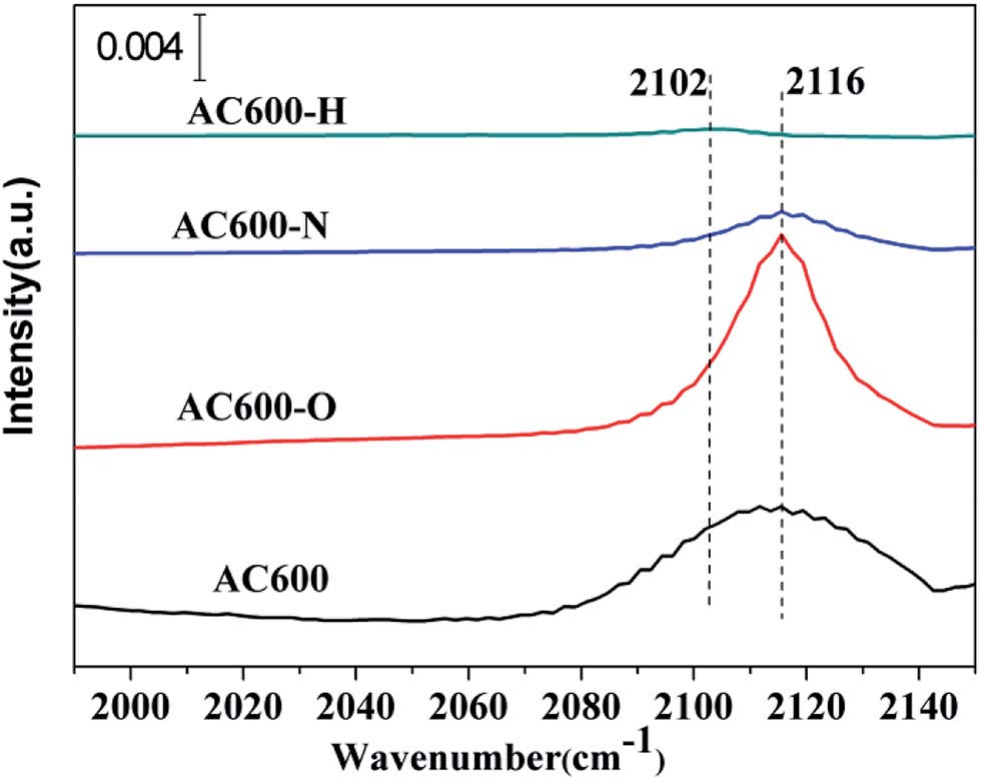
*In situ* DRIFT spectra of steady-state CO adsorption after 20 min on AC600, AC600-O, AC600-N and AC600-H at RT.

The *in situ* DRIFTS measurements of CO adsorption were recorded to investigate the CO adsorption change of the catalysts after the pretreatments, particularly to further examine the valance state of Au species and/or electron transfers. As shown in Fig. 8, a band is detected at 2106–2116 cm^−1^ in all the samples, which is ascribed to CO adsorbed at the metallic Au (CO–Au^0^).^25,26^ The CO–Au^0^ band for the unpretreated AC600 sample is centered at 2112 cm^−1^. Several important characteristics can be distinguished for the differently pretreated samples: (i) the peak intensity of the CO–Au^0^ band for AC600-O increased sharply. However, it decreased drastically after the N_2_ and H_2_ pretreatments. (ii) A red shift in the CO–Au^0^ band is only occurred for the AC600-H sample compared with that of the other samples. This indicates that there are more Au^0^ species in AC600-H samples, which is also an implication of the formation of the electron rich Au^0^. After purging with He, the CO adsorption peak decreased rapidly in intensity and disappeared completely (Fig. S5†). This result confirms that the metal-support interaction on the differently pretreated Au–CeO_2_ catalysts is weak. The impact of the size of the Au nanoparticles can be excluded since the size distribution of Au nanoparticles is similar in these four samples, which provides a good environment for us to study the interfacial contact between Au and CeO_2_. According to the previous reports, this can result from the lower CO adsorption sites primarily originating from the partial coverage of Au NPs by CeO_2_ supports after N_2_ and H_2_ pretreatments.^5^ Notably, AC600-N with the highest Au^0^ content may exhibit stable Au catalysis with high activity. This can be further verified by the Au dispersion test (Table S2†). The theoretical percentage of surface Au atoms (53.8%) was calculated from the average nanoparticle diameters. The percentage of surface Au atoms decrease in the following order: AC600 (14.6%) > AC600-O (13.1%) > AC600-N (12.9%) > AC600-H (11.9%). Thus, the Au NPs exist primarily on the CeO_2_ surface and are partially encapsulated.^27^ According to the *in situ* DRIFTS, XPS and HRTEM results, the coverage of CeO_2_ support on the Au NPs, the electron transfer and the changes in CO adsorption ability are in good agreement with the characteristics of metal-support interactions.

There are many efficient methods for altering the catalytic activities using oxidation or reduction pretreatments conditions. Moreover, inert (N_2_, He) pretreatments are often used to remove the surface impurities of the Au–CeO_2_ catalysts.^28^ However, in our study, it can be clearly observed that the catalytic performances of the Au–CeO_2_ calcined at 600 °C can be enhanced after mild N_2_ pretreatments. The construction of metal-support interaction can be proposed for AC600-N sample, where the coverage of CeO_2_ support on the Au NPs, the electron transfer and the changes in CO adsorption ability are in good agreement with those observed for metal-support interactions. The consecutive cycles of CO oxidation demonstrate that the metal-support interaction effect is real and reproducible. In addition, the AC600-H sample has poorer catalytic activity than the AC600-N sample, which may be due to the degree of encapsulation. However, a detailed mechanism still needs to be further discussed in the future.

## 4. Conclusion

In summary, we have displayed that the mild N_2_ pretreatments with the assistance of metal-support interactions could be a simple method to reactivate Au–CeO_2_ samples calcined at 600 °C. The achievement of the metal-support interaction results in a remarkable enhancement in CO oxidation activity, making it possible to obtain sintering-resistant Au catalysts. This study may provide a new understanding of the high catalytic stability of supported Au catalysts and can be extended to other sintering-resistant supported noble metal catalysts.

## Conflicts of interest

There are no conflicts to declare.

## Supplementary Material

RA-008-C8RA07278G-s001
