# Supplementary material for: Tuning the metal-support interaction in the thermal-resistant Au–CeO2 catalysts for CO oxidation: influence of a mild N2 pretreatment
Source: RSC Adv. 2018 Nov 23;8(68):39197–202. doi: 10.1039/c8ra07278g (PMC9090844; doi:10.1039/c8ra07278g)
Supplement: RA-008-C8RA07278G-s001 [file RA-008-C8RA07278G-s001.pdf]

Electronic Supplementary Material (ESI) for RSC Advances.
This journal is © The Royal Society of Chemistry 2018

Supporting Information

Tuning the metal-support interaction in the thermal-resistant Au-CeO_2 catalysts for CO oxidation: influence of one mild N_2 pretreatment

Yuqi Sun [a], Wei Liu *[a], Miao Tian [a], Liguo Wang [a], Zhongpeng Wang *[a]

[a]School of Water Conservancy and Environment, University of Jinan, Jinan 250022, China.

**Corresponding to: stu_liuw@ujn.edu.cn (Wei Liu); chm_wangzp@ujn.edu.cn (Zhongpeng Wang).*

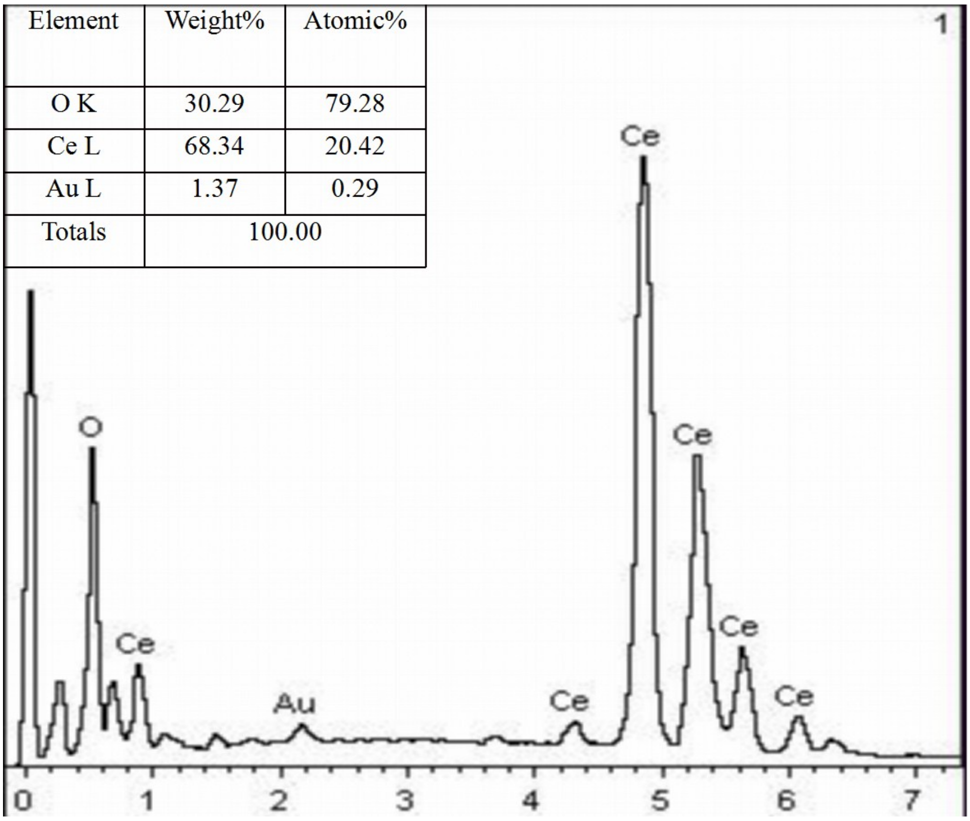

Element	Weight%	Atomic%
O K	30.29	79.28
Ce L	68.34	20.42
Au L	1.37	0.29
Totals	100.00	

Fig. S1 EDS spectrum of AC600 samples.

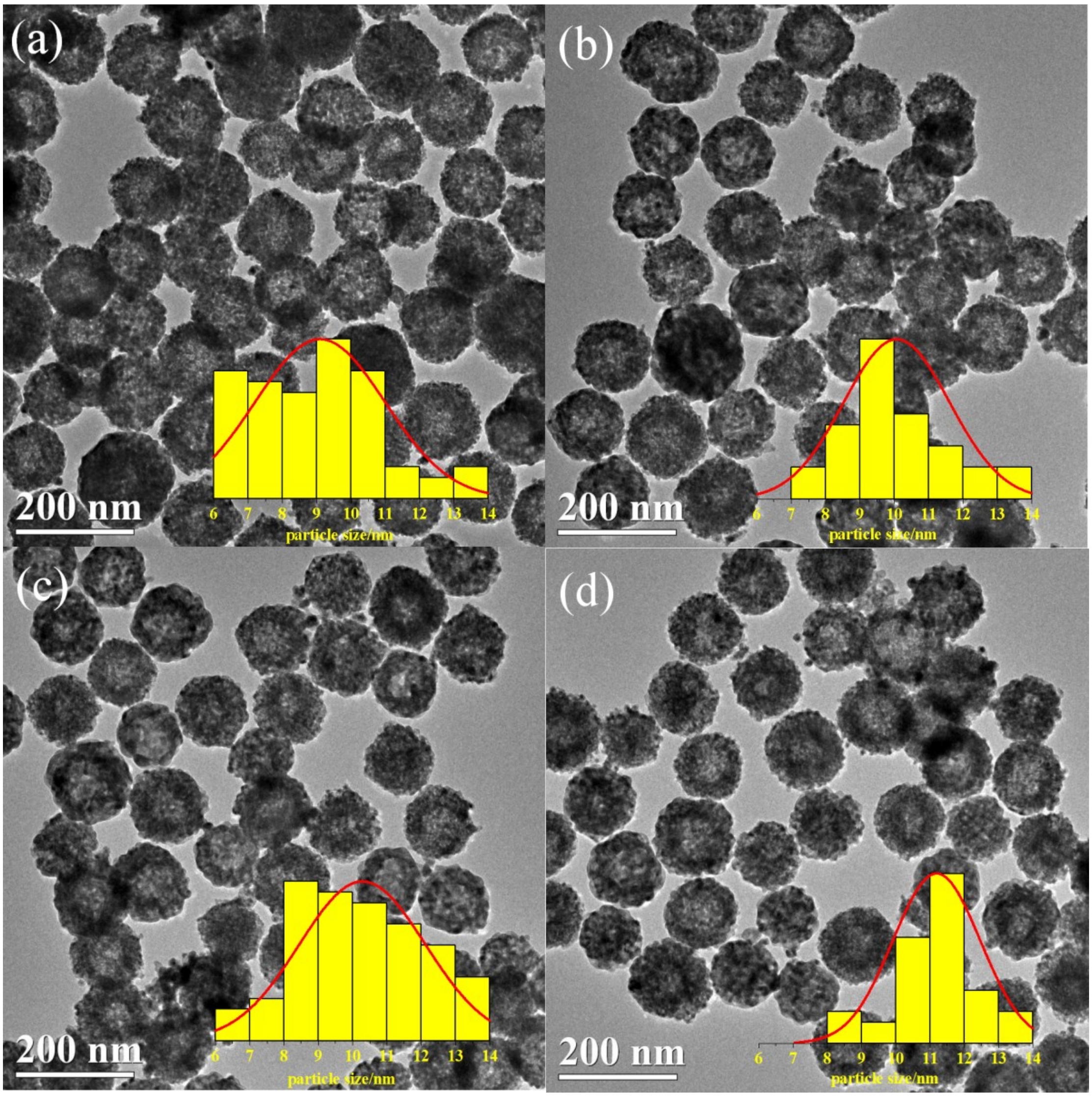

Fig. S2 TEM images and the corresponding size distribution of the different samples Au NPs. (a) AC600; (b) AC600-O; (c) AC600-N; (d) AC600-H.

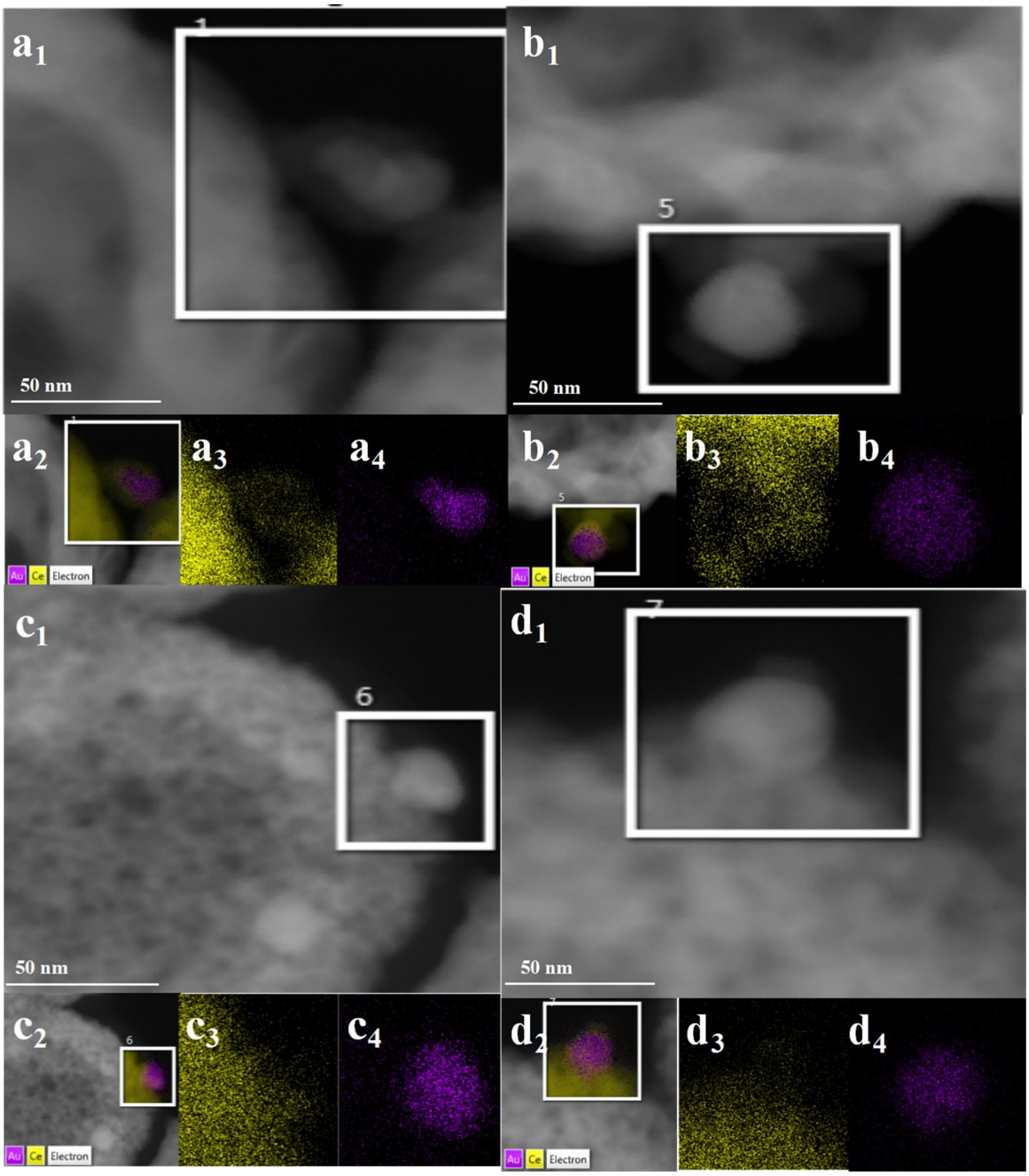

Fig. S3 STEM (x_1) and element mapping results (x_2, x_3, and x_4) of the AC600, AC600-O, AC600-N and AC600-H samples (x=a, b, c and d for AC600, AC600-O, AC600-N and AC600-H).

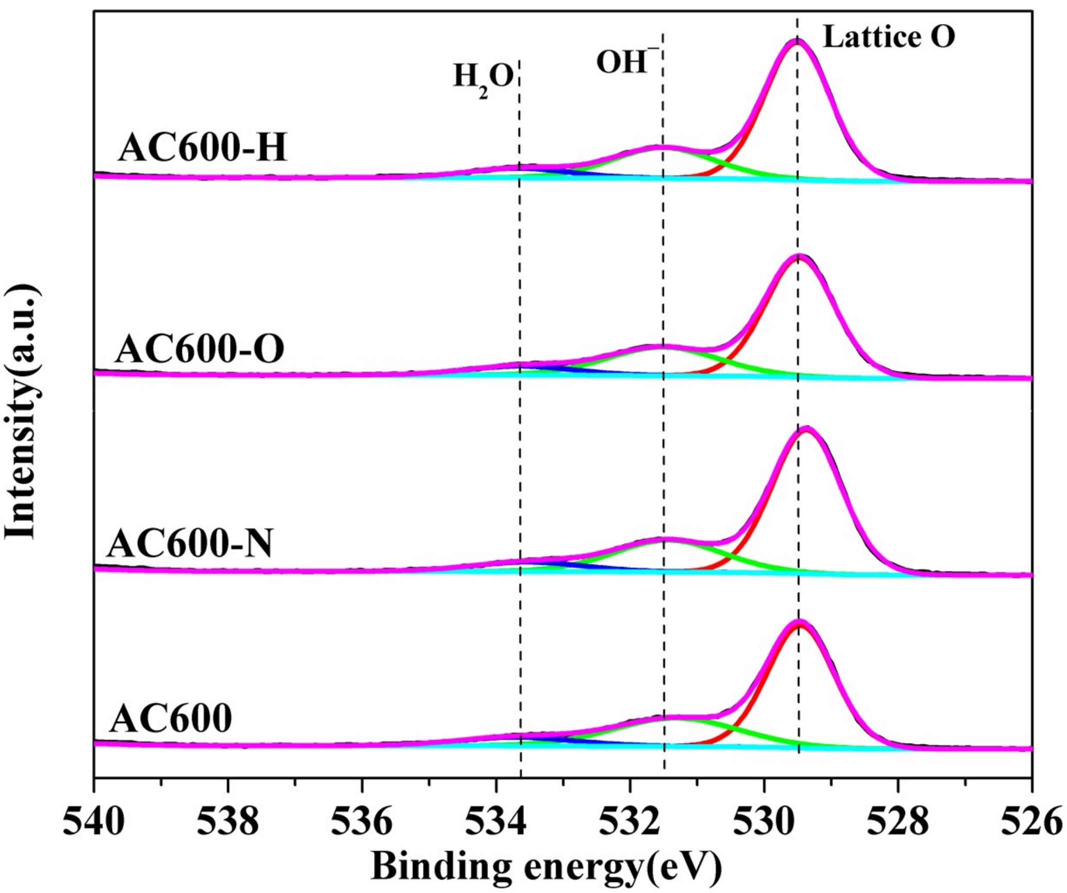

Fig. S4 XPS O1s spectra of AC600, AC600-N, AC600-O and AC600-H catalysts.

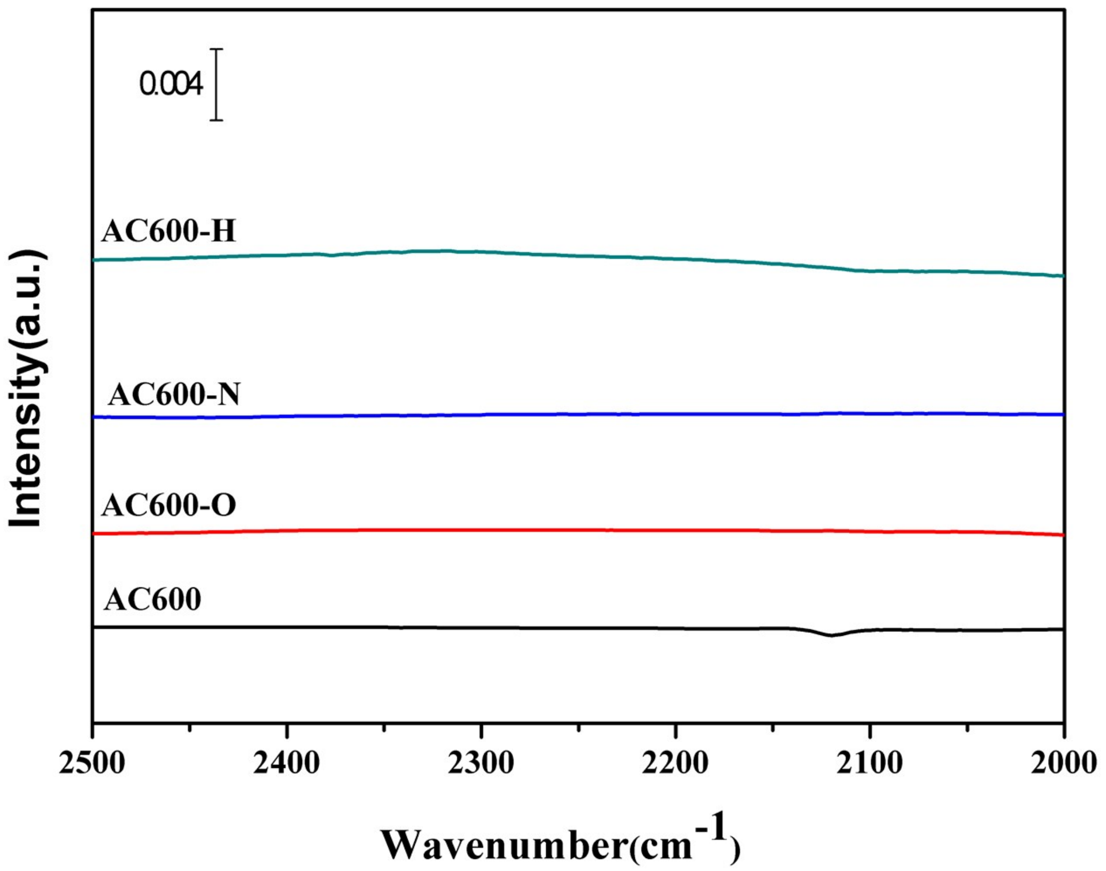

Fig. S5 The in situ DRIFT spectra of CO adsorption on AC600, AC600-O, AC600-N and AC600-H under the purge of He at room temperature.

Table S1 T_{50} and T_{100} for Au-CeO_2 pretreated in different atmospheres.

Samples	600°C	
	T_{50}	T_{100}
AC600	74	192
AC600-O	43	110
AC600-N	37	94
AC600-H	38	128

Table S2 Structural parameters of Au nanoparticles on various samples.

Samples	Average Au nanoparticle diameter (nm)	Percentage of surface Au atoms (%)[a]
AC600	9.1	14.6
AC600-O	10.1	13.1
AC600-N	10.3	12.9
AC600-H	11.2	11.9

[a]Calculated from the average Au nanoparticle diameters following the equation: Au dispersion = (1.33/Average nanoparticle diameter)*100%.

Table S3 Relative content of Au species for different catalysts obtained from Au4f XPS spectra.

Samples	Au^0			$Au^{\delta+}$		
	Peaks (eV)	content	FWHM (eV)	peaks	content	FWHM (eV)
AC600	84.3<br>87.8	50.7%	0.78	84.6<br>88.4	49.3%	2.06
AC600-O	84.3<br>87.8	51.8%	0.82	84.5<br>88.5	48.2%	1.90
AC600-N	84.4<br>88.1	71.3%	0.95	84.8<br>88.4	28.7%	2.32
AC600-H	84.2<br>87.9	76.2%	0.93	84.5<br>88.4	23.8%	2.02

Table S4 Relative content of Ce species for different catalysts obtained from Ce3d XPS spectra.

Catalysts	Ce^{3+}		Ce^{4+}	
	peaks	Content(%)	peaks	content(%)
AC600	885.1<br>902.5	18.4%	882.4, 889.1,<br>898.3, 900.8,<br>916.6, 907.4	81.6%
AC600-O	885.1<br>902.5	17.7%	882.4, 889.1,<br>898.3, 900.8,<br>916.6, 907.4	82.3%
AC600-N	885.2<br>902.5	18.0%	882.4, 889.1,<br>898.3, 900.8,<br>916.6, 907.4	82.0%
AC600-H	885.3<br>902.5	18.0%	882.4, 889.1,<br>898.3, 900.8,<br>916.6, 907.4	82.0%

Table S5 Relative content of oxygen species for different catalysts obtained from O1s XPS spectra.

Samples	lattice O		OH^-		H_2O	
	B.E. (eV)	Content(%)	B.E. (eV)	Content(%)	B.E. (eV)	Content(%)
AC600	529.5	64	531.3	28	533.7	8
AC600-O	529.5	67	531.5	26	533.6	7
AC600-N	529.4	69	531.4	24	533.5	7
AC600-H	529.5	69	531.5	24	533.7	7
